# Photo(chemo)therapy Reduces Circulating Th17 Cells and Restores Circulating Regulatory T Cells in Psoriasis

**DOI:** 10.1371/journal.pone.0054895

**Published:** 2013-01-24

**Authors:** Takuya Furuhashi, Chiyo Saito, Kan Torii, Emi Nishida, Sayuri Yamazaki, Akimichi Morita

**Affiliations:** Department of Geriatric and Environmental Dermatology, Nagoya City University Graduate School of Medical Sciences, Nagoya, Japan; Wayne State University, United States of America

## Abstract

**Background:**

Photo(chemo)therapy is widely used to treat psoriasis, the pathogenesis of which might be caused by an imbalance of Th17 cells/regulatory T cells (Treg). In the present study, we evaluated the effects of photo(chemo)therapy on the Th17/Treg balance and Treg function.

**Methods:**

Peripheral blood was obtained from psoriasis patients treated with bath-psoralen ultraviolet A (UVA, n = 50) or narrowband ultraviolet B (UVB, n = 18), and age-matched healthy volunteers (n = 20). CD3^+^CD4^+^IL-17A^+^ or CD4^+^CD25^+^Foxp3^+^cells were analyzed to estimate Th17 or Treg number by fluorescence–activated cell sorting. Moreover, CD4^+^ CD25^−^ T cells from patients treated with PUVA(n = 14) were incubated in CFSE and activated with or without CD4^+^ CD25^+^T cells, and the suppressive function of CD4^+^ CD25^+^T cells were analyzed.

**Results:**

Photo(chemo)therapy significantly reduced Th17 levels from 5.66±3.15% to 2.96±2.89% in patients with increased Th17 (Th17/CD4>3.01% [mean+SD of controls]). In contrast, photo(chemo)therapy significantly increased Treg levels from 2.77±0.75 to 3.40±1.88% in patients with less than 4.07% Treg level, defined as the mean of controls. Furthermore, while Treg suppressed the CD4^+^CD25^−^ T cell proliferation to a greater extent in controls (Treg Functional Ratio 94.4±4.28%) than in patients (70.3±25.1%), PUVA significantly increased Treg Functional Ratio to 88.1±6.47%. Th17 levels in severe patients (>30 PASI) were significantly higher as compared to controls. Th17 levels that were left after treatment in the patients not achieving PASI 50 (3.78±4.18%) were significantly higher than those in the patients achieving PASI 75 (1.83±1.87%). Treg levels in patients achieving PASI 90 (4.89±1.70%) were significantly higher than those in the patients not achieving PASI 90 (3.90±1.66%). Treg levels prior to treatment with Th17 high decreased group (5.16±2.20%) was significantly higher than that with Th17 high increased group (3.33±1.39%).

**Conclusion:**

These findings indicate that Treg is dysfunctional in psoriasis patients, and photochemotherapy restores those dysfunctional Treg. Photo(chemo)therapy resolved the Th17/Treg imbalance in patients with psoriasis.

## Introduction

Narrowband ultraviolet B (UVB, 311 nm) phototherapy is a popular treatment for refractory lesions such as those of psoriasis, atopic dermatitis (AD), and vitiligo [1]. Narrowband UVB is particularly effective for treating psoriasis, resulting in faster clearance of lesions, fewer episodes of excessive erythema, and a longer remission [2]. For psoriasis, the efficacy of narrowband UVB (311–313 nm) as compared to broadband UVB (290–320 nm) irradiation is due to the ability of 311-nm narrowband UVB to more effectively deplete skin-infiltrating T cells from the epidermis and dermis of psoriatic plaques [3]. Photochemotherapy with psoralen and UVA (PUVA) is widely used as an effective treatment for psoriasis. Although PUVA has become less popular, however, as narrowband UVB has become more popular, bath water delivery of 8-methoxypsoralen and subsequent UVA-irradiation (bath-PUVA therapy) remains an effective alternative to systemic application and the gold standard of photo(chemo)therapy modalities.

Phototherapy induces apoptosis as well as antigen-specific immunosuppression [4]. The narrowband UVB-induced depletion of pathogenically relevant T cells results from the induction of apoptosis [5]. Narrowband UVB therapy and bath-PUVA therapy generally induce a relatively long remission period of approximately 4 to 6 months in patients with psoriasis, a relatively long remission period that might be due only partly to the induction of apoptosis. The role of regulatory T cells (Treg) should also be considered, as narrowband UVB radiation induces local and systemic immune suppression in a model of contact hypersensitivity [6]. In patients with psoriasis, there is a functional defect in Treg suppressor activity that is not associated with a decrease in the number of CD25^+^ Treg in the peripheral blood [7]. In our previous clinical study [8], we examined whether bath-PUVA affects circulating Treg in the peripheral blood of psoriasis patients; 10 healthy controls and 18 psoriasis patients who had not previously received photo(chemo)therapy were enrolled. We assessed CD4^+^CD25^+^ (Forkhead box protein 3) Foxp3^+^ Treg in the peripheral blood of psoriasis patients before and after bath-PUVA therapy. Foxp3^+^Treg in peripheral blood mononuclear cells (PBMCs) tended to be lower in psoriasis patients (Treg/CD4; 4.57±2.40%) than in healthy volunteers (Treg/CD4; 6.00±1.39%) before bath-PUVA therapy, but increased significantly after bath-PUVA therapy in all patients (Treg/CD4; 6.40±2.85%). Bath-PUVA therapy also improved Psoriasis Area and Severity Index (PASI) scores and increased Foxp3^+^ Treg in all patients [8]. These findings indicate that bath-PUVA restores Treg in psoriasis patients, and suggest that the clinical efficacy of bath-PUVA therapy for psoriatic patients is due to the induction of Foxp3^+^ Treg. It is not known, however, whether photo(chemo)therapy restores Treg function.

T helper cells that produce interleukin (IL)-17 (Th17) are a newly characterized population of CD4^+^ effector T cells, distinct from Th1 and Th2 cells. A growing body of evidence indicates that Th17 cells are pathogenically related to psoriasis. An imbalance of Th17 cells and Treg is thought to contribute to the pathogenesis of psoriasis [9]. Therefore, in the present study, we evaluated the functional recovery of Treg and the contribution of Th17 to the effects of photo(chemo)therapy for psoriasis.

## Results

### Circulating Treg Levels in Patients with Psoriasis

**Figure 1:**
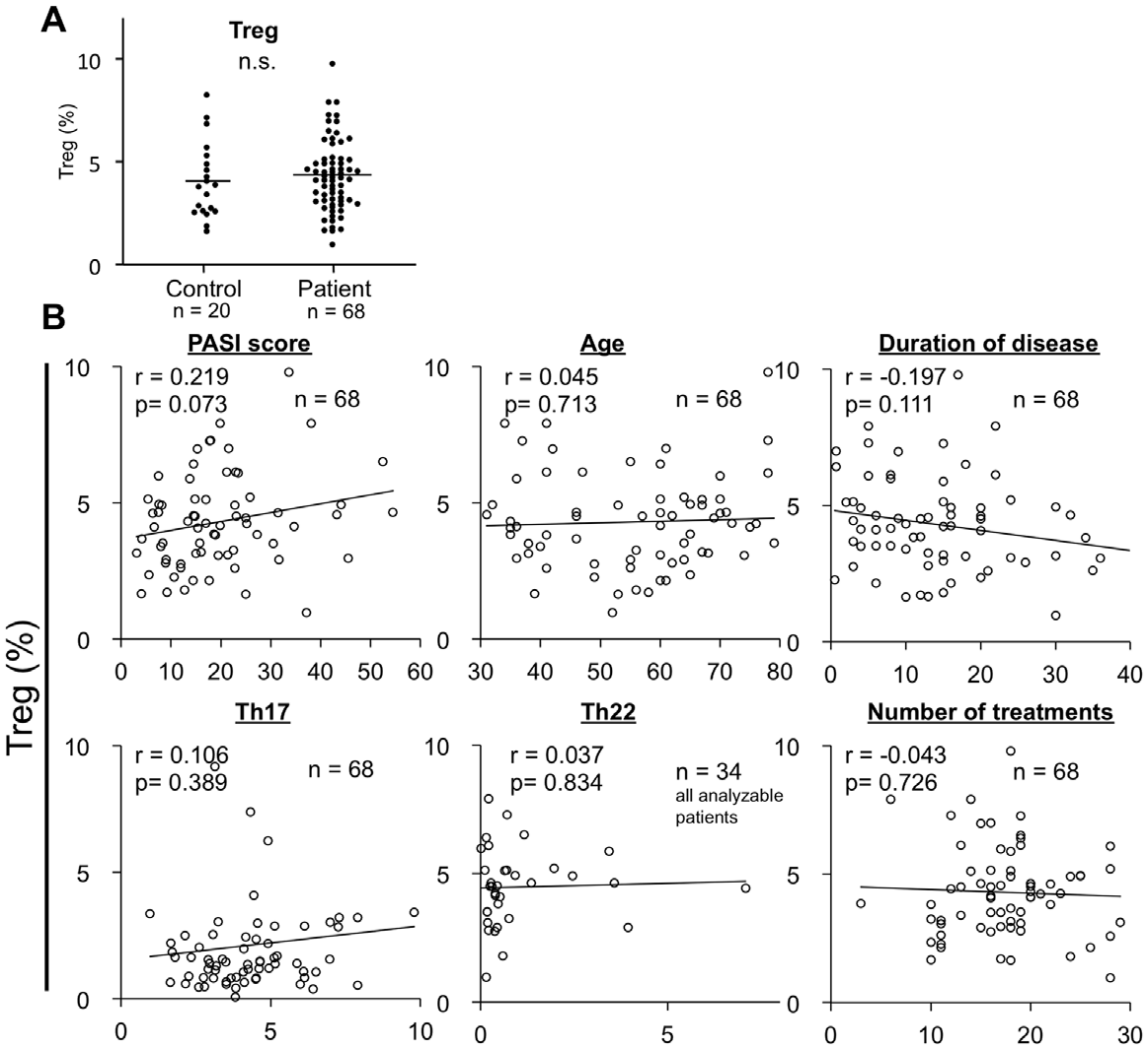
Circulating Treg levels in patients with psoriasis. (**A**) Dot plots show percentages of Treg in patients and controls. Horizontal bar indicates mean value. (**B**) The correlations between Treg levels and the other parameters, such as PASI score, age, disease duration, number of treatments and Th17, Th22 levels were examined.

To assess differences in the Treg levels between patients and controls, we compared the level of the population of CD4^+^CD25^+^Foxp3^+^T cells as Treg. The levels of Treg in the patients (4.29±1.73%, n = 68) was similar to those in the normal healthy controls (4.07±1.83% n = 20; Figure 1A). We next examined the correlation between Treg levels and the other parameters, such as PASI score, age, disease duration, number of treatments and Th17, Th22 levels. In this study, Treg levels were not correlated with any parameters. These results suggest that there might be something more important about Treg not only quantity.

### Photo(chemo)therapy Induces Increases Circulating Treg Concomitant with Clinical Efficacy

**Figure 2:**
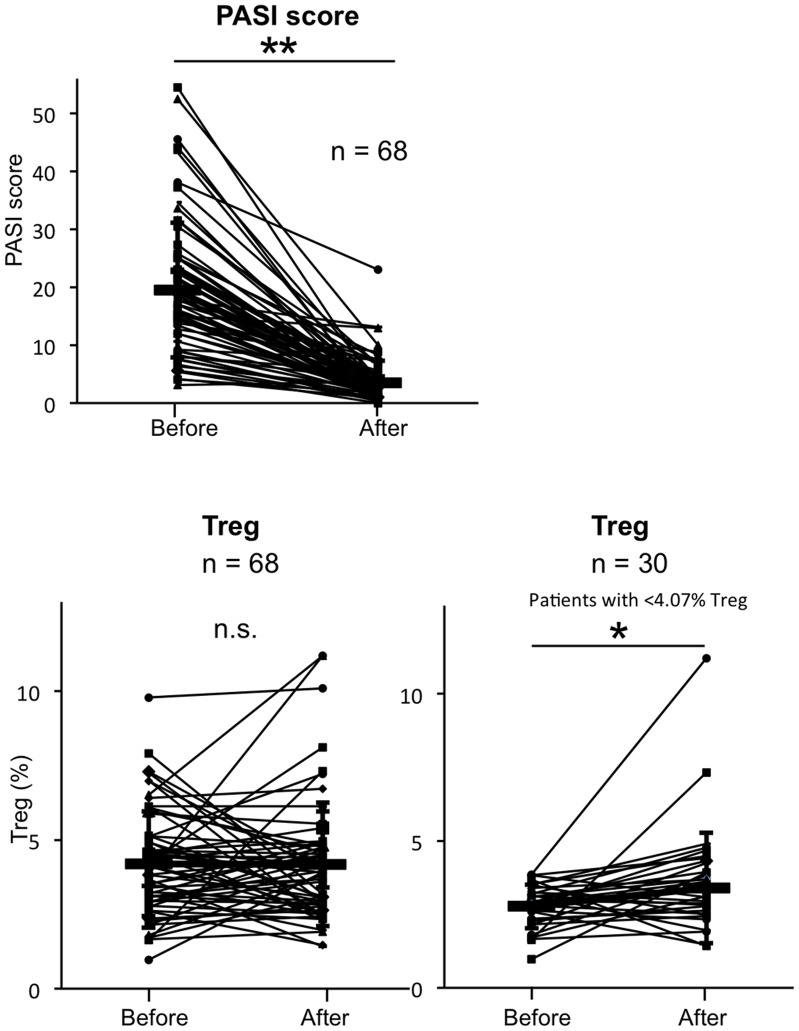
Photo(chemo)therapy increases circulating Treg population concomitant with clinical efficacy. Based on the PASI score, psoriasis lesions improved in all cases. Treg levels before and after photo(chemo)therapy in all patients and in the patients with less than 4.07% Treg, defined as the mean of controls, are shown. The results are presented as mean ± SD *p<0.05 and **p<0.01.

The psoriasis lesions were greatly improved in all cases (n = 68), based on PASI scores (from 19.5±11.6 to 3.50±3.82, p<0.01; Figure 2). Treg levels were compared before and after photo(chemo)therapy to confirm whether Treg increases were induced by photo(chemo)therapy (n = 68). Although Treg levels in all patients (n = 68) were not increased by photo(chemo)therapy (from 4.20±1.76 to 4.18±2.07%, p = 0.938), those in patients with less than 4.07% Treg, defined as the mean of controls, were significantly increased from 2.77±0.75 to 3.40±1.88% (p = 0.022, n = 30).

### Photochemotherapy Restores Dysfunctional Treg in Patients with Psoriasis

**Figure 3:**
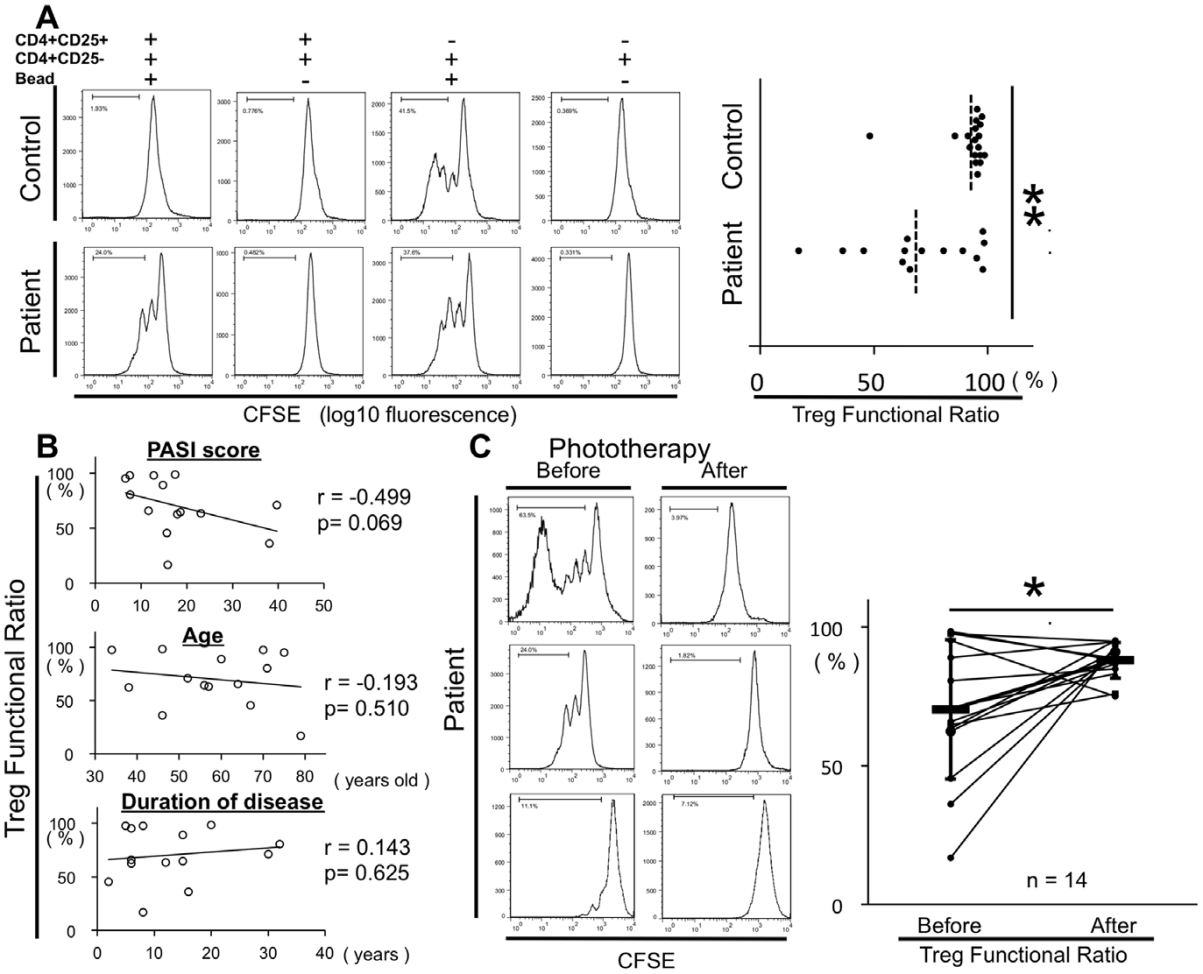
Photo(chemo)therapy restores dysfunctional Treg in patients with psoriasis. (**A**) CD4^+^T cells were separated with magnetic beads as CD25^+^T cells or CD25^−^ T cells. CD4^+^ CD25^−^ T cells were labeled with CFSE, and activated with anti-CD3, CD28 beads for 4 days, with or without CD4^+^ CD25^+^T cells (Treg) (CD4^+^ CD25^−^ T cells : CD4^+^ CD25^+^T cells = 2×10^5^ : 1×10^3^). FACS data for a typical patient and control are presented. Cells were gated on CD4^+^T cells. Treg did not suppress CD4^+^ CD25^−^ T cell proliferation in patients (lower panels) as effectively as in controls (upper panels). The ability of Treg to suppress the proliferation was referred to as the Treg Functional Ratio, determined by comparing the rate of the proliferated CD4^+^CD25^−^ T cells with and without CD4^+^CD25^+^T cells (Figure S1). Summarized data of Treg Functional Ratio in the patients (n = 14) and in the age-matched controls (n = 11) are shown. *p<0.05 and **p<0.01. Horizontal bar indicates mean value. (**B**) Correlation coefficient between Treg Functional Ratio and PASI score, age or disease duration (n = 14). (**C**) A Treg functional assay was performed with PBMC in patients before and after photochemotherapy. Histograms of the proliferated CD4^+^CD25^−^ T cells with CD4^+^CD25^+^T cells in three typical patients before and after treatment are shown. Summarized data of Treg Functional Ratio in the patients (n = 14) are shown. Treg Functional ratio in patients is restored to almost normal levels. *p<0.05.

Because we didn’t find the differences in circulating Treg levels between patients and controls, and the correlation with any other parameters, we focused not only level of Treg but also dysfunction of Treg in psoriasis patients. To confirm previous reports that psoriasis patients have dysfunctional Treg, we assayed Treg function (Figure 3A). PBMCs from patients were separated into CD4^+^ T cells, and this cell population was then separated into CD4^+^CD25^+^T cells and CD4^+^CD25^−^ T cells with magnetic beads. The isolated CD4^+^CD25^−^ T cells were labeled with CFSE, and activated with anti-CD3, CD28 beads for 4 days, with or without CD4^+^CD25^+^T cells (CD4^+^ CD25^−^ T cells: CD4^+^CD25^+^T cells = 2×10^5^ cells: 1×10^3^ cells). Although CD4^+^CD25^−^ T cells without CD4^+^CD25^+^T cells proliferated greatly in controls and underwent cell division about 3 to 5 times in 4 days, CD4^+^CD25^−^ T cells with CD4^+^CD25^+^T cells were suppressed and underwent, at most, one cell division. In contrast, CD4^+^CD25^−^ T cells with CD4^+^CD25^+^ T cells were not suppressed in psoriasis patients and underwent cell division 3 to 5 times, like the ratio of CD4^+^CD25^−^ T cells without CD4^+^CD25^+^T cells. We determined the ability of Treg to suppress CD4^+^CD25^−^ T proliferation as the Treg Functional Ratio by comparing the rate of the proliferating CD4^+^CD25^−^ T cells with and without CD4^+^CD25^+^T cells (Figure S1). The Treg Functional Ratio was significantly lower in the patients (70.3±25.1% n = 14) than in the age-matched controls (94.4±4.28% n = 18, p<0.01; Figure 3A). The Treg Functional Ratio was also inversely correlated with the PASI score (r = −0.499 p = 0.069), although it was not correlated with age or disease duration (Figure 3B). To evaluate whether photochemotherapy restored Treg function, we compared the Treg Functional Ratio before and after photochemotherapy (Figure 3C). The Treg Functional Ratio was significantly increased, and therefore Treg function was restored to almost normal levels (from 70.3±25.1 to 88.1±6.47%, p = 0.030). These results suggest that photochemotherapy not only increased the Treg number, but also restored Treg function. To confirm CD127 expression of CD4^+^CD25^+^T cells isolated, those cells in patients (n = 3) and controls (n = 3) were stained with anti-CD127 (Figure S2).

### Photo(chemo)therapy Induces a Decrease in Circulating Th17

**Figure 4:**
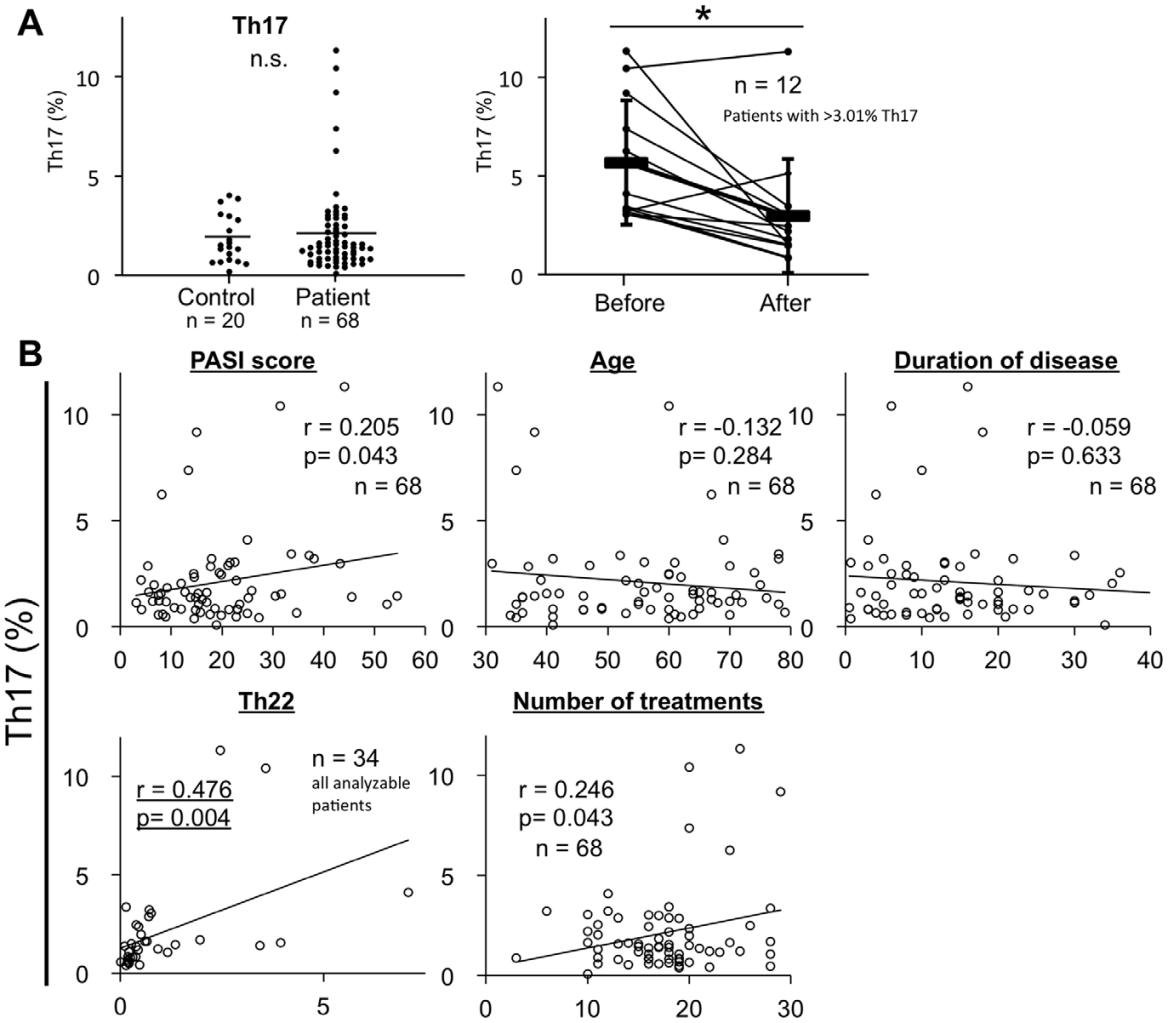
Photo(chemo)therapy decreases circulating Th17. (**A**) Dot plots show percentages of Th17 in patients and controls. Horizontal bar indicates mean value. Levels of Th17 greater than 3.01%, defined as the mean +1 SD of the control level, was analyzed. The higher levels of Th17 are shown, and those are restored to almost normal levels. *p<0.05. (**B**) Correlation coefficient between Th17 levels and PASI score, age, disease duration, the number of required treatment and Th22 levels.

We next compared the population levels of CD3^+^CD4^+^IL-17A^+^ T cells as Th17. Mean Th17 levels tended to be higher in patients (2.12±2.19%, n = 68) than in controls (1.81±1.20%, n = 20), but the difference was not significant (Figure 4A). We also compared Th17 levels before and after photo(chemo)therapy. Patients with more than 3.01% Th17, defined as the mean +1 SD of controls, were defined as the high-Th17 population (n = 12). Although Th17 levels in all patients (n = 68) were not decreased by photo(chemo)therapy (from 2.12±2.19 to 2.13±2.22%, p = 0.971), Th17 levels in the high-Th17 population were significantly reduced (from 5.66±3.15% to 2.96±2.89, p = 0.011; Figure 4A). Th17 levels were significantly correlated with Th22 levels and slightly correlated with number of treatments required (r = 0.476, p = 0.004 n = 34 and r = 0.246, p = 0.043, n = 68; Figure 4B). Our results indicated that photo(chemo)therapy bring Th17 level back to normal level in high-Th17 population, and Th17 levels prior to treatment might imply therapy resistance to photo(chemo)therapy.

### Th17 Level is Higher in Severe and Poor-responder Patients, Treg Level is Higher in Responder Patients

**Figure 5:**
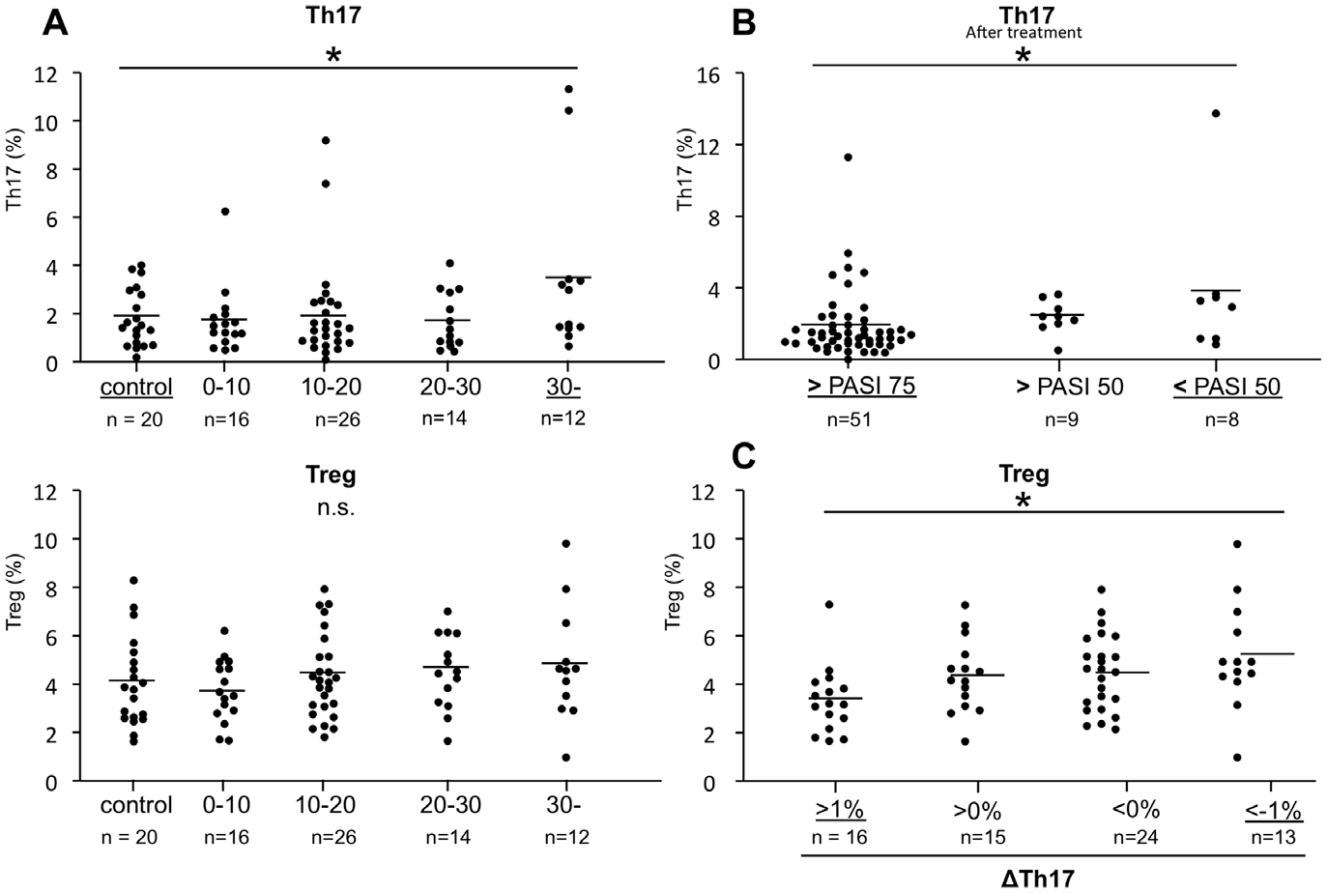
Th17 level is higher in severe and poor-responder patients, Treg level is higher in responder patients. (**A**) Psoriasis patients were grouped according to PASI score: 0≦PASI<10; 10≦PASI<20; 20≦PASI<30; 30≦PASI. Dot plots show the levels of Th17 or Treg in each group. Th17 levels in the patients with more than 30 PASI were significantly higher as compared to controls. Horizontal bar indicates mean value. (**B**) Psoriasis patients were grouped in each treatment group achieving 50%, 75% improvement PASI score from baseline (PASI 50, PASI 75). Dot plots show the levels of Th17 after treatment in each group and controls. Th17 levels after treatment in the patients not achieving PASI 50 were significantly higher than those in the patients achieving PASI 75. (**C**) Psoriasis patients were grouped according to the amount of decrease in Th17 levels by photo(chemo)therapy: Th17 high increased group n = 16, ΔTh17>1%; Th17 increased group n = 15, ΔTh17>0%; Th17 decreased group n = 24, ΔTh17<0%; Th17 high decreased group n = 13, ΔTh17<−1%. Dot plots show the levels of Treg prior to treatment in each group and controls. The levels of Treg prior to treatment with Th17 high decreased group; ΔTh17<−1% (5.16±2.20%) were significantly higher than that with Th17 high increased group; ΔTh17>1% (3.33±1.39%).

Next, to compare the levels of Th17 and Treg in each severity range, psoriasis patients were subdivided according to PASI score: 0≦PASI<10, n = 16; 10≦PASI<20, n = 26; 20≦PASI<30, n = 14; 30≦PASI, n = 12. The levels of Th17 in the patients with more than 30 PASI (3.52%±3.57 n = 12) were significantly higher than those in the control (1.81%±1.20 n = 20; Figure 5A). The means of Treg were gradually increased in the patients with increased PASI scores as follows, 0–10 (3.72%±1.26%), 10–20 (4.31±1.75%), 20–30 (4.50±1.53%), and >30 (4.79±2.36%), but not significant increased than in controls (4.07±1.83%). Next, psoriasis patients were subdivided in each treatment group achieving 50% and 75% improvement PASI score (PASI 50 and PASI 75). The levels of Th17 cells that were left after treatment in the patients not achieving PASI 50 (3.78±4.18%) were significantly higher than those in the patients achieving PASI 75(1.83±1.87%) (Figure 5B). In contrast, Treg levels prior to treatment in the patients achieving 90% improvement PASI score (PASI90) (4.89±1.70%) were significantly higher than those in the patients not achieving PASI 90 (3.90±1.66%) (data not shown). Finally, we estimated the amount of decrease in Th17 levels by photo(chemo)therapy. The patients were grouped according to ΔTh17: Th17 high increased group n = 16, ΔTh17>1%; Th17 increased group n = 15, ΔTh17>0%; Th17 decreased group n = 24, ΔTh17<0%; Th17 high decreased group n = 13, ΔTh17<−1%. The levels of Treg prior to treatment with Th17 high decreased group (5.16±2.20%) was significantly higher than that with Th17 high increased group (3.33±1.39%) (Figure 5C). In terms of clinical implication, these findings suggest that the patient with high Th17 level might be a severe patient or poor-responder, and the patient with high Treg level might be a therapy responder.

## Discussion

Treg are considered to be important for peripheral tolerance and for preventing autoimmune diseases [10]. The quantity and quality of Treg might be related to the pathogenesis of psoriasis. Therefore, we evaluated whether Treg number is altered in psoriasis patients. In a previous study, we found no difference in the percentage of Treg in psoriasis patients and that in healthy controls [8]. In contrast, however, another author reported that the frequencies of circulating Treg are higher in patients with severe psoriasis than in normal controls [9]. In that study, the number of enrolled patients was 54. In the present study, we analyzed the number of Treg in a similar number of patients. Mean Treg levels were not significantly higher in patients than in normal healthy controls. Based on our independent studies, the number of Treg in psoriasis patients is similar to that in healthy controls. We also checked whether CD4^−^ T cells expressed FoxP3, and found that CD4^−^ Foxp3^+^ cells were very small population in peripheral blood (Figure S3). Interestingly, Treg levels prior to treatment in the patients achieving PASI 90 were significantly higher than those in the patients not achieving PASI 90, and Treg levels with Th17 high decreased group were significantly higher than that with Th17 high increased group. Treg level might have a potential to predict whether responder or poor-responder. In our preliminary study, however, we observed recurrence even in patients in whom Treg levels were increased, suggesting that Treg number alone is not sufficient to explain the activity and pathogenesis of psoriasis. Therefore, we next evaluated Treg function. Long et al. suggested that not only Treg number, but also Treg function, should be considered in autoimmune disorders such as systemic lupus erythematosus, type 1 diabetes, and multiple sclerosis [11]. Sugiyama et al. reported that Treg are dysfunctional in psoriasis patients; however, they only analyzed three patients [7]. To further evaluate this possibility, we repeated the functional assay for Treg in 14 psoriasis patients. The functional levels of Treg were decreased in psoriasis patients. Finally, we demonstrated that photochemotherapy significantly increases the Treg Functional Ratio, restoring it to almost normal levels.

As narrowband UVB radiation induces local and systemic immune suppression in a model of contact hypersensitivity, the role of Treg should be considered [6]. The induction of apoptosis might be only partially responsible for the relatively long remission period achieved by narrowband UVB or bath-PUVA therapy. Treg cells have an immunoregulatory function and play a key role in peripheral tolerance [12]. Peripheral T cells from patients receiving UVB phototherapy exhibit a CD4^+^CD25^+^ T cell profile [13]. The induction of Treg cells following UV irradiation is associated with UV-induced DNA damage. UV-induced DNA damage induces Langerhans cells to move from the skin into the draining lymph nodes, and IL-12 can induce DNA repair and limit the number of UV-damaged Langerhans cells in the draining lymph nodes [14]. It is thus possible that UV-induced DNA damage alters cutaneous antigen-presenting cells and enhances their ability to activate Treg cells. UV irradiation increases the proportion of fluorescein isothiocyanate-bearing dendritic cells within the draining lymph nodes [15] that exhibit deficient maturation and deficient T cell priming [16]. UV irradiation, however, also promotes the generation of CD4^+^CD25^+^Foxp3^+^ T cells within the draining lymph nodes following immunization [17]. The induced CD4^+^CD25^+^Foxp3^+^ T cells are antigen-specific with regulatory function *in vivo*. These findings suggest that CD4^+^CD25^+^Foxp3^+^ T cells, i.e., Treg cells, are responsible for the immunosuppressive effects. In psoriasis, there is a functional defect in Treg suppressor activity that is not associated with a decrease in the number of CD25^+^ Treg in the peripheral blood [11].

A growing body of evidence suggests that CD4^+^Th17 contribute to the pathogenesis of psoriasis [18]. Plasmacytoid dendritic cells (pDCs), which are key regulators of the human antiviral and antibacterial immune response, are recruited to psoriatic lesions [19]. The activated pDCs produce interferon-α, which stimulates tumor necrosis factor-α and inducible nitric oxide synthase-producing DCs (Tip-DC) to produce IL-23 and IL-20 [20]. Dendritic cell activation and the production of IL-23 support Th17 cell survival and proliferation, and induce the production of IL-17 and IL-22 [21]. Th17 cells producing IL-17, IL-22, and tumor necrosis factor-α are pathogenically related to psoriasis. In a three-dimensional human epidermis model, IL-22 and IL-20 cause psoriasis-like morphologic changes associated with the inhibition of keratinocyte terminal differentiation and STAT3 upregulation [22]. Tip-DC and Th17 products activate keratinocytes, thereby promoting the release of innate inflammatory molecules [23], such as S100A7, defensins, and IL-8. In particular, the antimicrobial peptide LL37 forms aggregated condensed structures with DNA that are delivered to and retained within early endocytic compartments in pDCs [24]. In this study, Th17 levels were correlated with number of treatments required and IL-22^+^ T cell levels related to psoriatic severity. Moreover, in our preliminary study, Th17 levels in the patients received frequent phototherapy tended to higher than that in the patients who had never received. Therefore, we might predict whether a patient were resistant for treatment such as phototherapy.

An imbalance of Th17 cells and Treg is thought to contribute to the pathogenesis of psoriasis. In a clinical study, 14 patients with moderate to severe psoriasis were treated with narrowband UVB. Narrowband UVB suppresses the IL-23/IL-17 pathways, including IL-12/23p40, IL-23p19, IL-17, and IL-22, in normalized plaques, but not in nonresponsive plaques [25]. In another study, gene expression profiling was performed using epidermal RNA from lesional and nonlesional skin undergoing narrowband UVB. The Th17 pathway was downregulated during narrowband UVB in psoriatic epidermis [26]. In our previous study, photo(chemo)therapy significantly reduced the increased serum levels of both IL-17 and IL-22 in psoriasis patients compared to those in healthy volunteers. Furthermore, the percent reduction of the PASI correlated with serum levels of IL-6, but not IL-17 or IL-22, before photo(chemo)therapy [27], indicating that psoriasis patients with high serum IL-6 levels might be more susceptible to the effects of photo(chemo)therapy. Based on the findings of the present study, photo(chemo)therapy might suppress serum IL-17 and IL-22 levels by inhibiting the IL-6 induced generation of Th17. Kagami et al. reported that circulating Th17 levels are increased in psoriasis patients [28]. We further demonstrated that bath-PUVA therapy induced circulating Treg in patients with psoriasis [8]. It has remained unclear, however, whether Treg function is restored by phototherapy. We recently reported that 308-nm excimer light also induced Treg in patients with palmoplantar pustulosis [29]. In the present study, we clearly demonstrated that phototherapy increased the number of Treg and restored Treg function. Furthermore, the circulating Th17 levels were significantly decreased by both PUVA and narrowband UVB in patients with Th17 levels higher than 3.0%. Interestingly, the levels of Th17 cells that were left after treatment in the patients not achieving PASI 50 were significantly higher than those in the patients achieving PASI 75 and controls. This suggests that the remaining Th17 cells might cause therapy resistance. Photo(chemo)therapy induced a decrease in Th17 in the peripheral blood of psoriasis patients, thereby resolving the Th17 and Treg imbalance in these patients.

Natural sun exposure is sometimes recommended for inflammatory skin diseases. Heliotherapy is a well-described therapeutic modality and its potent effect involves immunoregulatory functions. In a human study, 20 patients with moderate to severe psoriasis were subjected to controlled sun exposure [30]. Both CD4^+^ and CD8^+^ T cells were significantly reduced in the epidermis and dermis of lesional skin. In contrast, dermal Treg levels were relatively increased, but the increase was not statistically significant. In the peripheral blood, skin-homing cutaneous lymphocyte-associated antigen positive T cells were significantly decreased after only 1 day in the sun and *in vitro*-stimulated PBMCs showed a reduced capacity to secrete cytokines, i.e., tumor necrosis factor-α, IL-12p40, IL-23p19, and IL-17a, after 16 days [30]. These findings indicate that sun exposure rapidly reduces local and systemic inflammation, and suggest that natural sunlight has a beneficial wavelength to induce immunosuppression for immunologic disorders. Besides natural sunlight, narrowband UVB and bath-PUVA therapy can be used to treat inflammatory skin diseases, including psoriasis. In the clinical setting, PUVA is superior to biologics therapy for the treatment of patients with mild to severe psoriasis [31].

Several studies have reported that Treg function was inversely correlated with CD127 expression in human [32]–[34] and mouse model of psoriasis [35]. Dysfunction of Treg in psoriasis might be related to level of CD127 expression in CD4^+^CD25^+^T cells separated in this study, and the restoration of dysfunctional CD4^+^CD25^+^T cells might be result of induction of CD127^low^ CD4^+^CD25^+^T cells by photochemotherapy.

Recently, some review papers providing new insight into Th17 have published, such as human cancer-associated immunity [36], Th17 expressing CD161 and interleukin-4-induced gene 1; Th17 function markers [37] and humanized anti-IL-17A monoclonal antibody [38]. Th17 might have a huge potential for a clue to pathogenesis of autoimmune and inflammatory diseases or cancer, and a target of treatment.

Taken together, CD4^+^CD25^+^T cells in psoriasis patients are dysfunctional, and photochemotherapy restores those dysfunctional cells to the normal level in this study. Th17 level is higher in severe and poor-responder patients, Treg level is higher in responder patients. Treg dysfunction and decreases in Treg levels are related to autoimmune and inflammatory disorders. Photo(chemo)therapy readily induces increases in Treg levels and restores Treg function, although the underlying mechanisms of Treg induction remain unclear. Photo(chemo)therapy may be applicable for systemic immune diseases in humans.

## Materials and Methods

### Study Design

All psoriasis vulgaris patients who were treated in the Phototherapy center of Department of Geriatric and Environmental Dermatology in Nagoya City University between February 2009 and December 2011. 50 patients were enrolled for Bath-PUVA therapy, 18 patients were enrolled for narrow-band UVB therapy, and age-matched 20 healthy volunteers were enrolled as controls. All patients enrolled in this open-label study were diagnosed with psoriasis clinically and histologically (Table S1). The patients were resistant to topical corticosteroids or topical vitamin D3 analogues. Patients continued to use moisturizers and the same topical treatments used before the study. Th22 analysis was performed between October 2009 and December 2011 and Treg functional assay was performed between March 2011 and December 2011. The study was approved by the Ethics Committee of Nagoya City University Graduate School of Medical Sciences, Nagoya, Japan. All subjects provided written informed consent.

### Photo(chemo)therapy Protocol

Patients were treated with 0.0001% psoralen baths for 15 min preceding treatment with UVA radiation 5 times weekly (starting UVA dose, 0.5 J/cm^2^; dose increment, 0.5 J/cm^2^). The maximum dose was 4.0 J/cm^2^. Peripheral blood was obtained from patients before bath-PUVA therapy and 24 h after the last exposure.

For narrowband UVB therapy, we determined the patient’s minimal erythema dose (MED) to establish the optimal dosage schedule prior to phototherapy. The initial dose should equal 0.5 MED. Patients were treated once or twice per week. If the initial dose was tolerated, the previous dose was increased by 20% at each visit. When a previous treatment resulted in erythema, no treatment was given the next day or the dose was decreased, depending on whether the erythema was asymptomatic or severe and painful. All patients tolerated both the bath-PUVA and narrowband UVB.

### Assessment

PASI scores were assessed before and after treatment. Clinical photographs were obtained and assessed independently by two of the authors (T.F. and C.S.).

### Peripheral Blood Samples

After obtaining written informed consent, PBMCs were obtained from 68 psoriasis patients before and after treatment. For Treg, PBMCs were stained with antibodies against CD4, CD25 (DAKO, Carpinteria, CA), and Foxp3 (eBioscience, San Diego, CA), and then subjected to fluorescence-activated cell sorting analysis (FACS Calibur™, Becton Dickinson, Franklin Lakes, NJ). The frequency of CD4^+^CD25^+^Foxp3^+^/CD4^+^cells, which were defined as Treg cells, was evaluated. For Th17 analysis, CD4^+^T cells were isolated using magnetic beads (Miltenyi Biotec, Auburn, CA), incubated with phorbol 12-myristate 13-acetate and ionomycin (Sigma Aldrich, St. Louis, MO) for 4 h in the presence of brefeldin A (BD Bioscience, San Jose, CA), and stained with a monoclonal antibodies against CD3 (BD Bioscience, eBioscience), IL-17A (eBioscience) and IL-22(R&D Systems, Minneapolis, MN). CD3^+^IL-17A^+^ T cells and CD3^+^IL-22^+^ T cells were defined as Th17, Th22 cells, measured by fluorescence-activated cell sorting analysis (FACS Calibur™). The frequency of IL-17^+^CD3^+^CD4^+^/CD3^+^CD4^+^ cells and IL-22^+^CD3^+^CD4^+^/CD3^+^CD4^+^ cells were investigated as Th17, Th22 cells.

### Functional Assay

PBMCs were separated as CD4^+^CD25^+^T cells or CD4^+^CD25^−^ T cells with CD4^+^CD25^+^ using the Regulatory T cell Isolation Kit (Miltenyi Biotec). CD4^+^ CD25^−^ T cells were incubated in 10 µM carboxyfluorescein diacetate succinimidyl ester (CFSE, Invitrogen Life Technologies, Paisley, UK) at 37°C for 10 min and activated with anti-CD3, CD28-bound beads (Miltenyi Biotec) for 4 days, with or without CD4^+^ CD25^+^T cells (CD4^+^ CD25^−^ T cells:CD4^+^ CD25^+^T cells = 2×10^5^∶1×10^3^) in serum-free X-VIVO15 medium (Lonza, Basel, Switzerland). We determined the ability of Treg to suppress CD4^+^ CD25^−^ T proliferation as the Treg Functional Ratio by comparing the rate of the proliferated CD4^+^CD25^−^ T cells with and without CD4^+^CD25^+^T cells (Figure S1). CD4^+^ CD25^+^T cells separated with MACS beads were stained with anti-CD127 (Biolegend, San Diego, USA) (Figure S2). The purity of CD4^+^ CD25^+^T cells was more than 90%.

### Statistical Methods

Statistical analyses were performed using Pharmaco Analyst II (Humanlife, Tokyo, Japan). We compared the levels of Treg, Th17 and Treg Functional Ratio between in patients and controls using student’s *t-*test. PASI score, the levels of Th17 and Treg of each patient at baseline and after each treatment using the paired *t*-test. Almost all correlation analyses were performed to establish the relationship among some parameters using the Pearson product-moment correlation coefficient, only the correlation between the Treg functional ratio and PASI score was performed using the Spearman’s rank Correlation Coefficient. The Th17 and Treg levels of patients grouped with PASI were compared using Williams’ test (Figure 5A). The Th17 and Treg levels of grouped patients with PASI50, 75, 90 and according to ΔTh17 were compared using Dunnett’s test (Figure 5B, C). The results are presented as the mean±SD. A p value of less than 0.05 was considered significant.

## Supporting Information

Figure S1 **The Treg Functional Ratio is calculated as shown in the figure. “a” is the rate of the proliferated CD4^+^CD25^−^T cells cultured with CD4^+^CD25^+^T cells.** In contrast, “b” is the rate of proliferated CD4^+^CD25^−^T cells cultured without CD4^+^CD25^+^T cells. This ratio indicates the extent to which Treg suppresses the proliferation of effector T cells.
(TIFF)

Figure S2 **PBMCs from three patients and three controls were separated for CD4^+^CD25^+^T cells using MACS beads.** CD4^+^CD25^+^T cells were stained with CD4, CD25, CD127. FACS plots were gated on CD4^+^T cells.
(TIFF)

Figure S3 **PBMCs from a patient and control were stained for CD4 and FoxP3.**
(TIF)

Table S1 **This table is showing all psoriasis patients and controls enrolled in this study.**
(DOCX)
