# Supplementary figures and images for: Photo(chemo)therapy Reduces Circulating Th17 Cells and Restores Circulating Regulatory T Cells in Psoriasis
Source: PLoS One. 2013 Jan 24;8(1):e54895. doi: 10.1371/journal.pone.0054895 (PMC3554687; doi:10.1371/journal.pone.0054895)

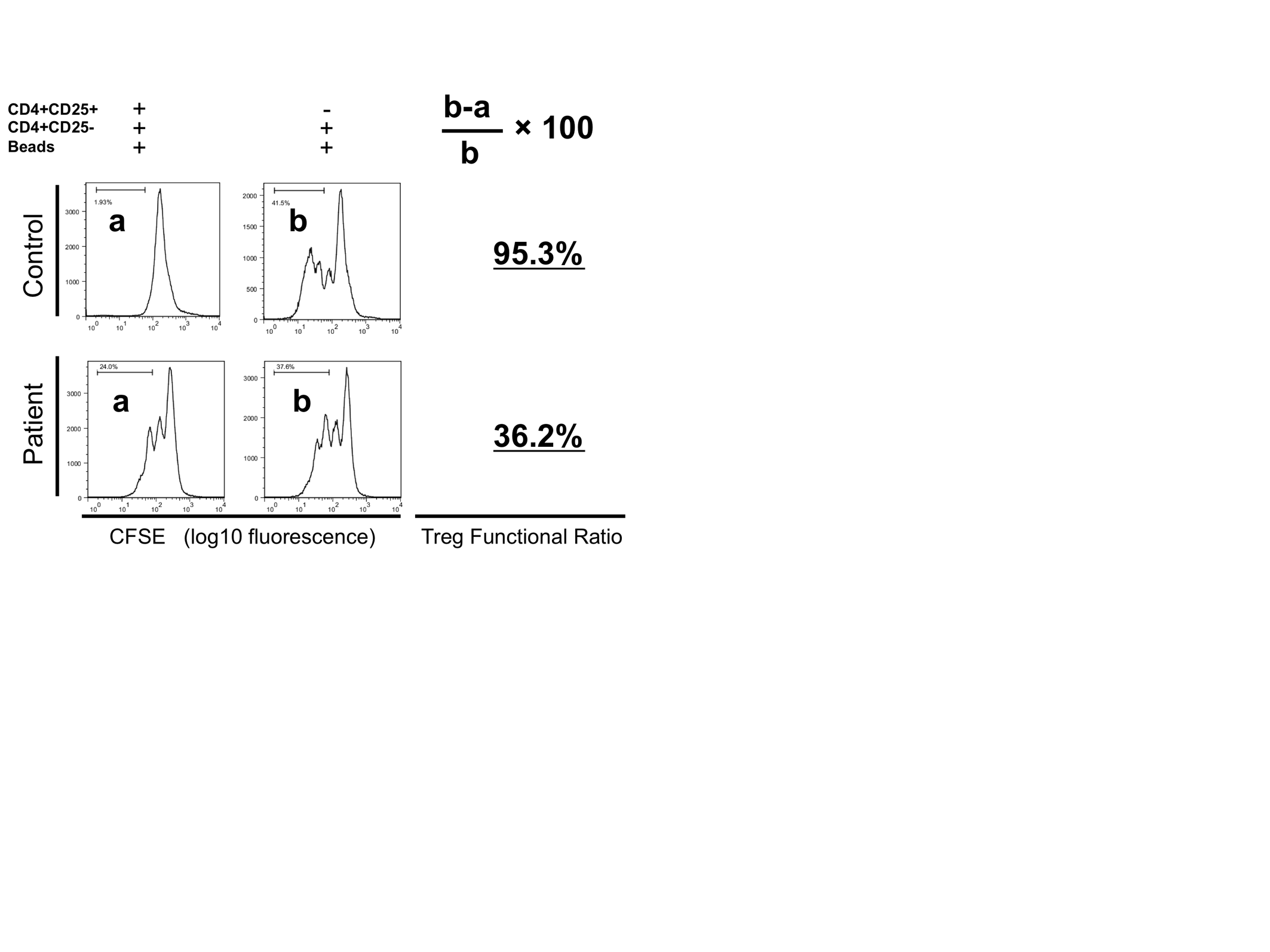

Supplement: Figure S1 — The Treg Functional Ratio is calculated as shown in the figure. “a” is the rate of the proliferated CD4+CD25−T cells cultured with CD4+CD25+T cells. In contrast, “b” is the rate of proliferated CD4+CD25−T cells cultured without CD4+CD25+T cells. This ratio indicates the extent to which Treg suppresses the proliferation of effector T cells. (TIFF) [file pone.0054895.s001.tiff]

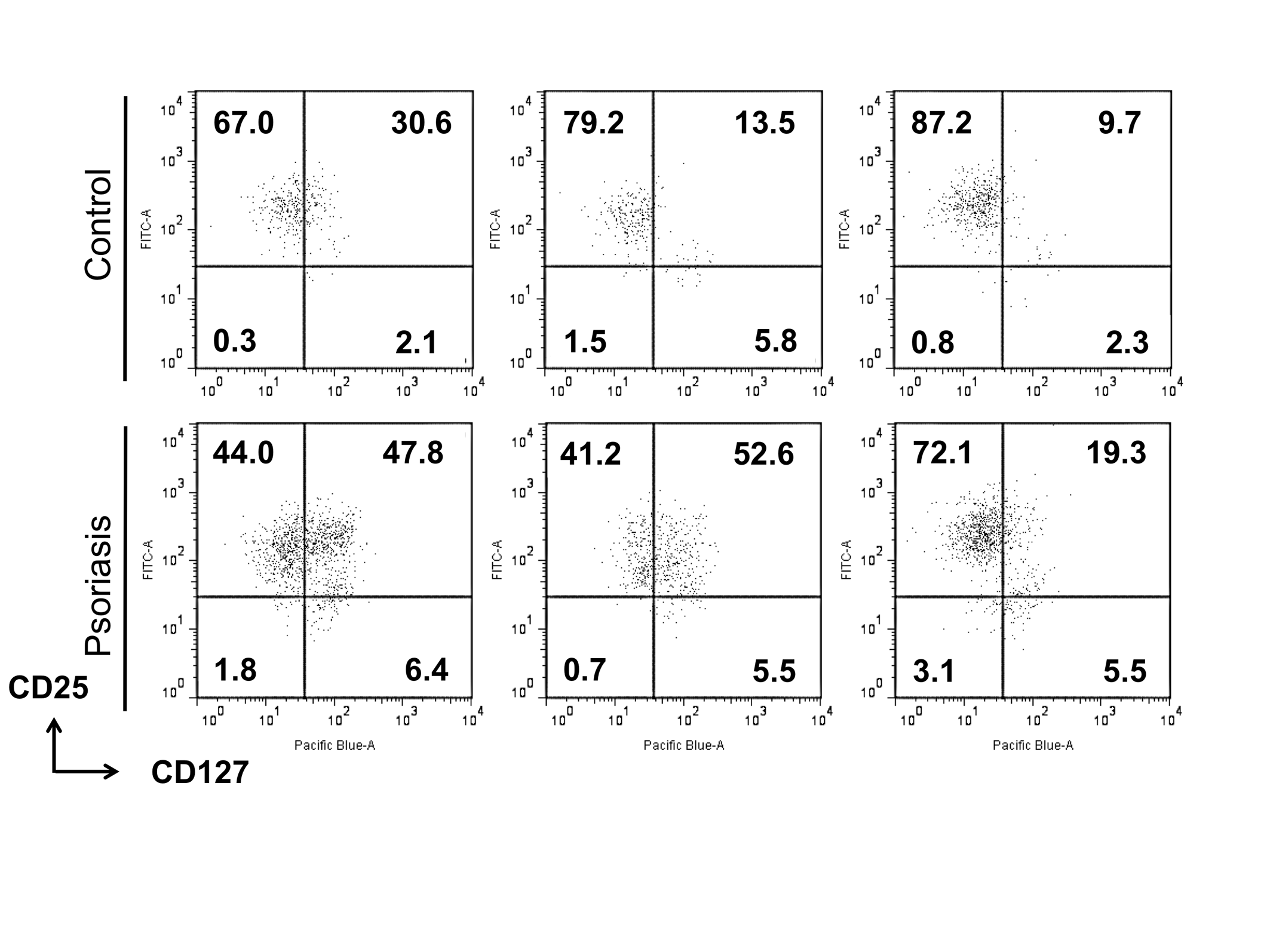

Supplement: Figure S2 — PBMCs from three patients and three controls were separated for CD4+CD25+T cells using MACS beads. CD4+CD25+T cells were stained with CD4, CD25, CD127. FACS plots were gated on CD4+T cells. (TIFF) [file pone.0054895.s002.tiff]

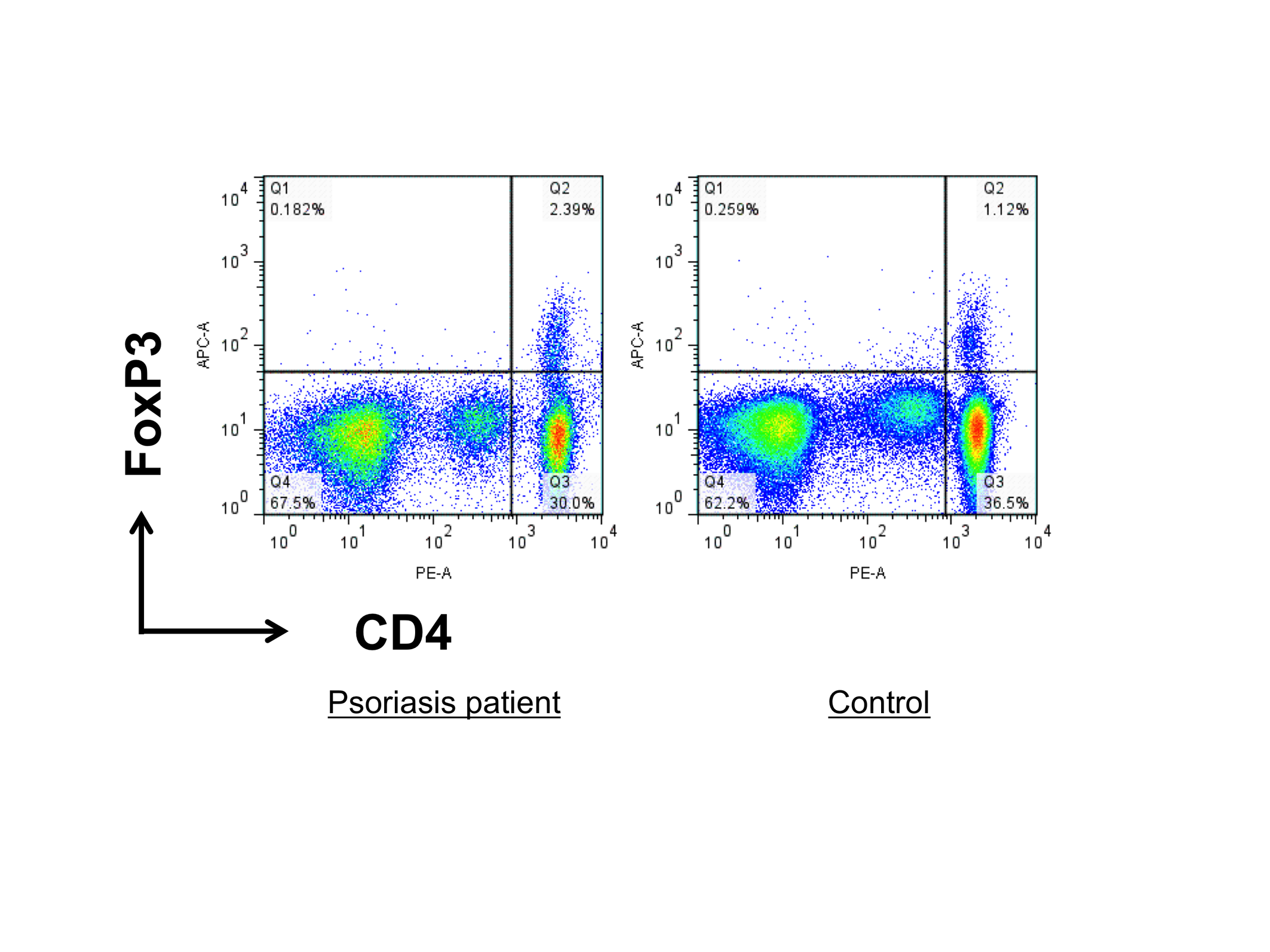

Supplement: Figure S3 — PBMCs from a patient and control were stained for CD4 and FoxP3. (TIF) [file pone.0054895.s003.tif]
