# Supplementary material for: Photo(chemo)therapy Reduces Circulating Th17 Cells and Restores Circulating Regulatory T Cells in Psoriasis
Source: PLoS One. 2013 Jan 24;8(1):e54895. doi: 10.1371/journal.pone.0054895 (PMC3554687; doi:10.1371/journal.pone.0054895)
Supplement: Table S1 — This table is showing all psoriasis patients and controls enrolled in this study. (DOCX) [file pone.0054895.s004.docx]

**Table S1. Patient Characteristics**

|  | **Bath-PUVA^1^** | **Narrowband UVB^2^** | **Controls** |
| --- | --- | --- | --- |
| Number of cases | 50 | 18 | 20 |
| Mean age | 55.8 ± 14.3 | 53.8 ± 12.8 | 51.2 ± 9.3 |
| Male:Female | 40 : 10 | 15 : 3 | 7 : 13 |
| Mean PASI^1^ score | 21.3 ± 12.0 | 14.4 ± 9.0 | NA^4^ |

^1^PUVA, psoralen with ultraviolet A

^2^ UVB, ultraviolet B

^3^ PASI, Psoriasis area severity index

^4^ NA, not applicable
